# Ibogaine Administration Modifies GDNF and BDNF Expression in Brain Regions Involved in Mesocorticolimbic and Nigral Dopaminergic Circuits

**DOI:** 10.3389/fphar.2019.00193

**Published:** 2019-03-05

**Authors:** Soledad Marton, Bruno González, Sebastián Rodríguez-Bottero, Ernesto Miquel, Laura Martínez-Palma, Mariana Pazos, José Pedro Prieto, Paola Rodríguez, Dalibor Sames, Gustavo Seoane, Cecilia Scorza, Patricia Cassina, Ignacio Carrera

**Affiliations:** ^1^Departamento de Histología y Embriología, Facultad de Medicina, Universidad de la República, Montevideo, Uruguay; ^2^Laboratorio de Síntesis Orgánica, Departamento de Química Orgánica, Facultad de Química, Universidad de la República, Montevideo, Uruguay; ^3^Departamento de Neurofarmacología Experimental, Instituto de Investigaciones Biológicas Clemente Estable, Montevideo, Uruguay; ^4^Department of Chemistry, Columbia University, New York, NY, United States

**Keywords:** ibogaine, neurotrophic factors, GDNF, BDNF, NGF

## Abstract

Ibogaine is an atypical psychedelic alkaloid, which has been subject of research due to its reported ability to attenuate drug-seeking behavior. Recent work has suggested that ibogaine effects on alcohol self-administration in rats are related to the release of Glial cell Derived Neurotrophic Factor (GDNF) in the Ventral Tegmental Area (VTA), a mesencephalic region which hosts the soma of dopaminergic neurons. Although previous reports have shown ibogaine’s ability to induce GDNF expression in rat midbrain, there are no studies addressing its effect on the expression of GDNF and other neurotrophic factors (NFs) such as Brain Derived Neurotrophic Factor (BDNF) or Nerve Growth Factor (NGF) in distinct brain regions containing dopaminergic neurons. In this work, we examined the effect of ibogaine acute administration on the expression of these NFs in the VTA, Prefrontal Cortex (PFC), Nucleus Accumbens (NAcc) and the Substantia Nigra (SN). Rats were i.p. treated with ibogaine 20 mg/kg (I_20_), 40 mg/kg (I_40_) or vehicle, and NFs expression was analyzed after 3 and 24 h. At 24 h an increase of the expression of the NFs transcripts was observed in a site and dose dependent manner. Only for I_40_, GDNF was selectively upregulated in the VTA and SN. Both doses elicited a large increase in the expression of BDNF transcripts in the NAcc, SN and PFC, while in the VTA a significant effect was found only for I_40_. Finally, NGF mRNA was upregulated in all regions after I_40_, while I_20_ showed a selective upregulation in PFC and VTA. Regarding protein levels, an increase of GDNF was observed in the VTA only for I_40_ but no significant increase for BDNF was found in all the studied areas. Interestingly, an increase of proBDNF was detected in the NAcc for both doses. These results show for the first time a selective increase of GDNF specifically in the VTA for I_40_ but not for I_20_ after 24 h of administration, which agrees with the effective dose found in previous self-administration studies in rodents. Further research is needed to understand the contribution of these changes to ibogaine’s ability to attenuate drug-seeking behavior.

## Introduction

Ibogaine is the main indole alkaloid isolated from the root bark of the African shrub *Tabernanthe iboga* (Lavaud and Massiot, 2017). Traditionally used in African religious ceremonies as a psychedelic, ibogaine became a subject of interest to the scientific community due to its reported ability to reduce craving and self-administration of several drugs of abuse in humans (Brown, 2013). These effects found mainly in uncontrolled clinical trials and observational studies, have been reported to be long-lasting enduring weeks to months after a single administration of large doses of ibogaine (Schenberg et al., 2014; Brown and Alper, 2017; Noller et al., 2017; Corkery, 2018; Malcolm et al., 2018; Mash et al., 2018). In animal models for drug dependence, ibogaine also reduces the self-administration of morphine and heroin (Glick et al., 1991, 1994; Dworkin et al., 1995), cocaine (Cappendijk and Dzoljic, 1993; Glick et al., 1994), and alcohol (He et al., 2005), with long-lasting effects that persists beyond pharmacokinetic elimination of the drug (Alper, 2001). In addition, ibogaine administration to animals also reduces naloxone or naltrexone precipitated-withdrawal signs (Dzoljic et al., 1988; Glick et al., 1992; Leal et al., 2003).

Although a vast amount of research has been done regarding the pharmacology of ibogaine, the mechanism of action of its ability to attenuate drug-seeking behavior remains unresolved (Alper, 2001; Maciulaitis et al., 2008; Brown, 2013). Ibogaine binds to numerous central nervous system (CNS) targets at the micromolar range such as: nicotinic acetylcholine receptors (nAChR α3β4 and α2β4) (Fryer and Lukas, 1999; Arias et al., 2010, 2015), N-methyl-D-aspartate (NMDA) (Mash et al., 1995b), kappa and mu opioid (Antonio et al., 2013; Maillet et al., 2015), 5HT_2A_ and 5HT_3_ receptors (Glick et al., 2000) and the dopamine and serotonin transporters (Mash et al., 1995a; Glick et al., 2001; Asjad et al., 2017). However, these ibogaine-receptor interactions do not seem to account for the long-lasting effects of ibogaine found in rodents which are described to last for 48 to 72 h after ibogaine administration (Glick et al., 1991, 1994; Cappendijk and Dzoljic, 1993). In rodents, ibogaine has a short half-life of 1–2 h raising the hypothesis that its longer-lived active metabolite, noribogaine, could be responsible for the enduring effects elicited by ibogaine. Both, the parent drug and its metabolite have differences in their binding profiles and affinities to the abovementioned CNS receptors (Staley et al., 1996). However, no appreciable amounts of noribogaine have been found in rodents’ brain tissue 19 h after ibogaine intraperitoneal (i.p.) administration (Pearl et al., 1997), and only approximately 5% of the noribogaine Cmax was detected in serum 24 h after the same treatment (Baumann et al., 2001b).

A few years ago, a novel hypothesis linking ibogaine’s attenuation of alcohol self-administration in rodents to its ability to modulate the expression of Glial Cell Derived Neurotrophic Factor (GDNF) in the brain was proposed. It was shown that a single ibogaine i.p. administration (40 mg/kg) increased the expression of GDNF in the midbrain of rats and mice for up to 24 h (He et al., 2005). In addition, microinjection of ibogaine into the Ventral Tegmental Area (VTA), produced a long-lasting reduction of ethanol self-administration, a response that was attenuated by the intra-VTA delivery of anti-GDNF neutralizing antibodies. These results suggested that ibogaine mediates its effects against ethanol consumption by increasing GDNF content in the VTA (He et al., 2005). Accordingly, another study from the same research group showed that the intra-VTA infusion of noribogaine induced a long-lasting decrease in ethanol self-administration (Carnicella et al., 2010). Further, ibogaine-derived synthetic derivatives were recently shown to induce the release of GDNF *in vitro*, in established cell line systems (Gassaway et al., 2016). These observations formed the basis for a new rationale to explain the long-lasting effects of ibogaine; i.e., the induction of GDNF by ibogaine/noribogaine may activate an autocrine loop, leading a long-term synthesis and release of GDNF (that persists beyond elimination of both substances). This mechanism may reverse the biochemical adaptations to chronic exposure to drugs of abuse in the reward system (He and Ron, 2006).

Neurotrophic Factors (NFs), such as GDNF and BDNF (Brain Derived Neurotrophic Factor) are small proteins that promote the growth, differentiation, synaptogenesis, and survival of neurons. Their expression in the nervous tissue is relatively high during the development of the CNS, where substantial growth, differentiation and remodeling of the nervous system occur (Barde, 1990; Lu and Figurov, 1997). More recently, it has been discovered that NFs play important roles in the adult brain where they modulate maintenance, protection, repair and plasticity of the nervous tissue (Reichardt, 2006; Schmidt and Duman, 2007). Furthermore, accumulating evidence has suggested that GDNF and BDNF mediate neuronal remodeling processes that occur during the development of substance use disorders (SUDs) (Bolaños and Nestler, 2004; McGough et al., 2004; Angelucci et al., 2007; Jeanblanc et al., 2009; Bie et al., 2012). Particularly, the role of GDNF and BDNF in the neuroadaptations in the mesocorticolimbic dopamine system (Prefrontal Cortex, PFC- VTA-Nucleus Accumbens, NAcc pathway) induced by repeated exposure to drugs of abuse has been extensively studied, including the impact of manipulating NFs levels on drug-seeking behavior in animal models (Russo et al., 2009; Ghitza et al., 2010; Koskela et al., 2017). It has been shown that the administration of BDNF or GDNF can either promote or inhibit drug-taking behaviors depending mainly on the brain site of administration, along with other several factors such as the drug type, the addiction phase (initiation, maintenance, abstinence or relapse), the time interval between site-specific NFs injections and the related behavioral assessments (Ghitza et al., 2010). For example, BDNF infusion into the NAcc increases cocaine-seeking behavior (Graham et al., 2007), while BDNF infusion into the medial pre-frontal cortex (mPFC) suppresses it (Berglind et al., 2007). Additionally, infusion of BDNF into the dorsolateral striatum decreases ethanol self-administration in rats (Jeanblanc et al., 2009).

**FIGURE 1:**
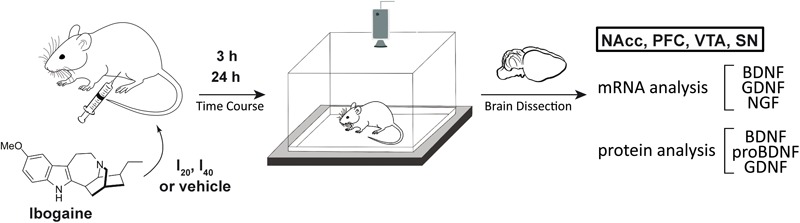
Schematic showing the experimental design of this work. Experimental groups of animals were i.p. treated with ibogaine 20 mg/kg (I_20_), 40 mg/kg (I_40_) or vehicle. After 3 and 24 h, locomotion of control and treated animals was recorded using an open field test. Afterward, animals were sacrificed, and selected brain regions were dissected. mRNA levels for BDNF, GDNF, and NGF were determined by qPCR. Western Blot was used to determine BDNF, proBDNF, and GDNF protein content. PFC = Prefrontal Cortex, NAcc = Nucleus Accumbens, VTA = Ventral Tegmental Area, and SN = Substantia Nigra, GDNF = Glial Cell Derived Neurotrophic Factor, BDNF = Brain Derived Neurotrophic Factor, NGF = Nerve Growth Factor.

Given the importance and the site-specificity of the elicited responses, we decided to analyze the effect of a single administration of ibogaine on the expression of GDNF and BDNF (mRNA transcripts and protein content) at two time points in those brain areas which define the mesocorticolimbic dopamine system such as VTA, PFC and NAcc (Figure 1). As the Substantia Nigra (SN) is a major nucleus of dopaminergic neurons important in the basal ganglia functioning, the expression of these NFs in this region was also studied. In order to examine the impact of ibogaine administration on the expression of other relevant NFs (which impact on drug-seeking behaviors has been much less studied) the Nerve Growth Factor (NGF) transcript content was also analyzed in the abovementioned brain areas. Selected time points were chosen considering previous pharmacokinetics reports in rats using i.p. administration (Pearl et al., 1997; Zubaran et al., 1999; Baumann et al., 2001a,b). In this manner, we chose to study NFs expression/content in the selected brain areas at 3 h, where ibogaine and noribogaine are present in relevant concentrations (Baumann et al., 2001b), and at 24 h where ibogaine is no longer detected and no significant amounts of noribogaine would be present in the brain (Pearl et al., 1997). In this manner, is expected that the observed effects found at 24 h, would be due to long lasting mechanisms elicited by the drug which remain after it has been cleared from the brain, but not from the acute effects of ibogaine/noribogaine. Finally, a behavioral study recording the locomotor activity of the control and drug-treated animals was performed using an open field test for each time point.

## Materials and Methods

### Ibogaine HCl

The ibogaine used in this study was chemically synthesized using voacangine as starting material, which was extracted from the root bark of *Voacanga africana* (purchased from CAPE LABS) using a modification of a previously described procedure (Jenks, 2002). Briefly, 100g of grounded root bark of *V. africana* was extracted with a 1% aqueous solution of HCl (6 × 500 mL). The combined aqueous extracts were basified by adding concentrated NH_4_OH until pH 10–11. A brown precipitate was separated by centrifugation and dried at 60°C for 24 h. This solid was taken in acetone and filtered to discard root impurities. The solvent was evaporated *in vacuo* to afford a total alkaloid extract of 3.5–4.0 g. Column chromatography (SiO_2_, Hex:EtOAc:NH_4_OH, 90:10:0.01) allowed to obtain 1g of pure voacangine which was analyzed by ^1^H and ^13^C NMR (See Supplementary Material). Voacangine was decarboxylated as follows. To a solution of voacangine in EtOH (0.45 M) in a double necked round bottomed flask, KOH in pellets (5 equivalents) was added. The solution was heated to reflux until consumption of the starting material was evident by thin layer chromatography (TLC) analysis. EtOH was removed under reduced pressure, and the residue was dissolved at 0°C in a round bottomed flask using a 6% (v/v) aqueous solution of HCl (enough quantity to adjust pH to 1). The system was then heated to reflux for 5 min. Once the starting material consumption was evident by TLC analysis, the solution was carefully basified using 50% NaOH (pH 10–11). Precipitation of ibogaine as a white solid was observed. Ethyl acetate was added, and the resultant biphasic system was transferred into a separation funnel. The aqueous phase was extracted three times with EtOAc. The combined organic layers were dried under Na_2_SO_4_, and the solvent was removed *in vacuo*. Purification was carried out using column chromatography purification (SiO_2_, hexanes: ethyl acetate 8:2 + 0.5% ammonium hydroxide). Ibogaine free base was obtained with an 86% and was analyzed by ^1^H and ^13^C NMR (see Supplementary Material). Crystallization from EtOH afforded a crystalline solid which was converted to the corresponding hydrochloride by treatment with diethyl ether saturated with HCl(g). Purity of ibogaine⋅HCl was determined by GC-MS analysis as 98.3% (see Supplementary Material). Dissolution of ibogaine-HCl to prepare the samples for i.p. injection was carried out using warm saline that was previously degassed by nitrogen bubbling.

### Experimental Animals

Thirty-six male Wistar adult rats (270–300 g) were used in this study and assigned to one of the following groups: Vehicle group at 3 and 24 h (*n* = 6 per each group); Ibogaine 20- (I_20_) treated group at 3 and 24 h (*n* = 6 per each group) and Ibogaine 40- (I_40_) treated group at 3 and 24 h (*n* = 6 per each group). Animals were housed four to five per cage and maintained on a 12-h light/dark cycle (lights on at 07.00 h) with food and water freely available before and after i.p. injection of vehicle or ibogaine until behavioral testing and sacrifice. All experimental procedures were conducted in agreement with the National Animal Care Law (#18611) and with the “Guide to the care and use of laboratory animals” (8th edition, National Academy Press, Washington, DC, 2010). Furthermore, the local Institutional Animal Care Committee (IIBCE) approved the experimental procedures (Protocol Number 007/05/2014). Adequate measures were taken to minimize pain, discomfort or stress of the animals, and all efforts were made to use the minimal number of animals necessary to obtain reliable scientific data.

#### Behavioral Analysis

Animals were brought to the experimental room in their home cages, identified and weighed prior to the behavioral test. An open field (OF) apparatus consisting of a square area (45 cm wide × 45 cm long × 40 cm high) with transparent plastic walls indirectly illuminated (35 luxes) to avoid reflection and shadows were employed. The OF was placed in a quiet experimental room with controlled temperature (22 ± 2°C). As rats were not habituated to the OF before drug or vehicle administration, novelty-induced motor activity was automatically recorded by a camera connected to a computer equipped with the Ethovision XT 12.0 software (Noldus, Netherlands) located above the OF. Using this video tracking software, we specifically measured the total distance traveled in meters (m) during 30 min, starting 3 and 24 h after ibogaine or vehicle administration. Animals were randomly assigned to different experimental groups and were used only once. Taking into account that immediately after i.p. administration ibogaine can produce a dose-dependent unusual motor profile and some prototypical serotonergic syndrome-related behaviors (e.g., tremor, flat body posture, forepaw treading) (Gonzalez et al., 2018), these specific behaviors were assessed by a trained investigator every 5 min (for a total of 30 min) starting 3 and 24 h after ibogaine administration. During all experiments, the OF was cleaned with 30% alcohol before placing the following rat. All experiments were done between 9 AM and 3 PM.

### *Ex vivo* Studies

#### Brain Dissection

Three or twenty-four hours after I_20_, I_40_ or vehicle (i.p.) injection, animals were sacrificed by decapitation and the brains were carefully removed and chilled in ice cold saline. According to Paxinos and Watson (2005), the whole NAcc (shell and core), PFC (including mPFC), Substantia Nigra (SN, pars compacta-SNpc and pars reticulata-SNpr) and VTA were dissected out on ice and the tissue obtained was immediately frozen and rapidly stored at –80°C until the processing day (Scorza et al., 1997; Meikle et al., 2013). Representative examples of coronal section at the level of each dissected brain area are shown in the Supplementary Material.

#### Semiquantitative qPCR

For RT-PCR analysis total RNA was extracted from the different brain regions using Trizol reagent (Thermo Fisher Scientific) followed by chloroform extraction and isopropanol precipitation. Possible DNA contaminations were eliminated with DNase treatment using DNase free Kit (Thermo Fisher Scientific). RNA quality was evaluated by agarose gel electrophoresis followed by ethidium bromide staining and quantified using a NanoDrop 1000 Spectrophotometer (Thermo Fisher Scientific). 500 ng of this total RNA was reverse-transcribed using 200 U M-MLV-reverse transcriptase (Thermo Fisher Scientific) following manufacturer instructions. 25 ng of the resulting cDNA was diluted in Biotools Quantimix Easy master mix (Biotools) in 10 μl volume. All reactions were performed in triplicates in strip tubes (Axygen^®^ Brand Products), using specific forward and reverse primers. The sequences of the quantitative PCR primers (IDT, Integrated DNA Technologies) used are as follows: for GAPDH F: 5′-CAC TGA GCA TCT CCC TCA CAA-3′ and R: 5′-TGG TAT TCG AGA GAA GGG AGG-3′, for BDNF F: 5′-GAG GGG TAG ATT TCT GTT TGT T-3′ and R: 5′-TTG CCT TAA TTT TTA TTC GTT T-3′, for GDNF F: 5′-AAA TCG GGG GTG CGT CTT AAC T-3′ and R: 5′-AAC ATG GGC CTA CCT TGT C-3′, for NGF F: 5′-AAG TTA TCC CAG CCA AAC TA-3′ and R: 5′-ATG TCA GTG TTG GGA GTA GG-3′. According to the sample, we used cycles 15–23 (the threshold cycle, Ct), in order to calculate the relative amounts of our gene of interest. PCR amplification was done over 40 cycles using a Rotor-Gene 6000 System (Corbett Life Science) and data were analyzed using Rotor Gene 6000 software (Corbett Life Science). Quantification was performed with ΔΔCt method using rats treated with vehicle as a negative control, and GAPDH mRNA as reference.

#### Western Blot Analysis

The selected brain regions were sonicated in a lysis buffer containing 50 mM NaCl, 50 mM HEPES, 2 mM sodium orthovanadate, 1% Triton X-100, and SigmaFAST Protease inhibitor cocktail (Sigma-Aldrich). After quantification and denaturation, the samples were loaded and separated by 12% SDS-PAGE gels and then transferred into a nitrocellulose membrane. The membranes were incubated for 1 h in blocking solution (BS: 5% Bovine serum albumin, 1% Tween 20 in PBS), and incubated overnight at 4°C with primary antibodies to GDNF (1:500 in BS; Abcam ab119473), BDNF (1:400 in BS; Promega G1641), or proBDNF (1:500 in BS; Invitrogen PA1-18360), together with anti-alpha-tubulin (1:3000 in BS; Abcam ab184613) as loading control. Afterward, the membranes were washed and incubated for 1 h at room temperature with IRDye 680RD/IRDye 800CW-Conjugated Goat Anti-Mouse IgG/Goat Anti-Rabbit IgG/Donkey Anti-Chicken IgG secondary antibodies (1:15000 in PBS each, LI-COR Biosciences #926-68070, #926-32210, #926-68071, #926-32211, and #925-32218). The Odyssey system (LI-COR Biosciences) was used to detect the bands. Quantification of band intensity was performed using Image Studio software version 5.2.5.

### Data Analysis

GraphPad Prism software 5 was used to design figure graphs and data analysis. Data are presented as mean ± SEM values. Six animals per group were assessed for behavioral and PCR studies. In some cases, some data was excluded from the analysis due to insufficient sample or high deviation from the mean of the group, rendering a lower *n*, but never smaller than 4. The total sample size (*N*) is given in figure legends and the sample for each treatment (*n*) can be observed in the scatter plot graphs in each figure. For western blot analysis, samples from 4 animals per group were assessed. Data from qPCR and western blot were analyzed and compared by one-way ANOVA followed by *post hoc* Tukey’s Multiple Comparison Test. In all cases, statistical significance was set at *P* < 0.05. General *P* and *F* values from ANOVA, and *p* values from Tukey’s multiple comparison test are provided in figure legends for each data set when significance is reached. Also, eta squared values (η^2^) accounting for effect size are provided. Data from motor activity were analyzed by two-way (treatment, time, and interaction between factors) ANOVA for repeated measures followed by Newman–Keuls multiple comparison *post hoc* test and Unpaired-*t*-test.

## Results

**FIGURE 2:**
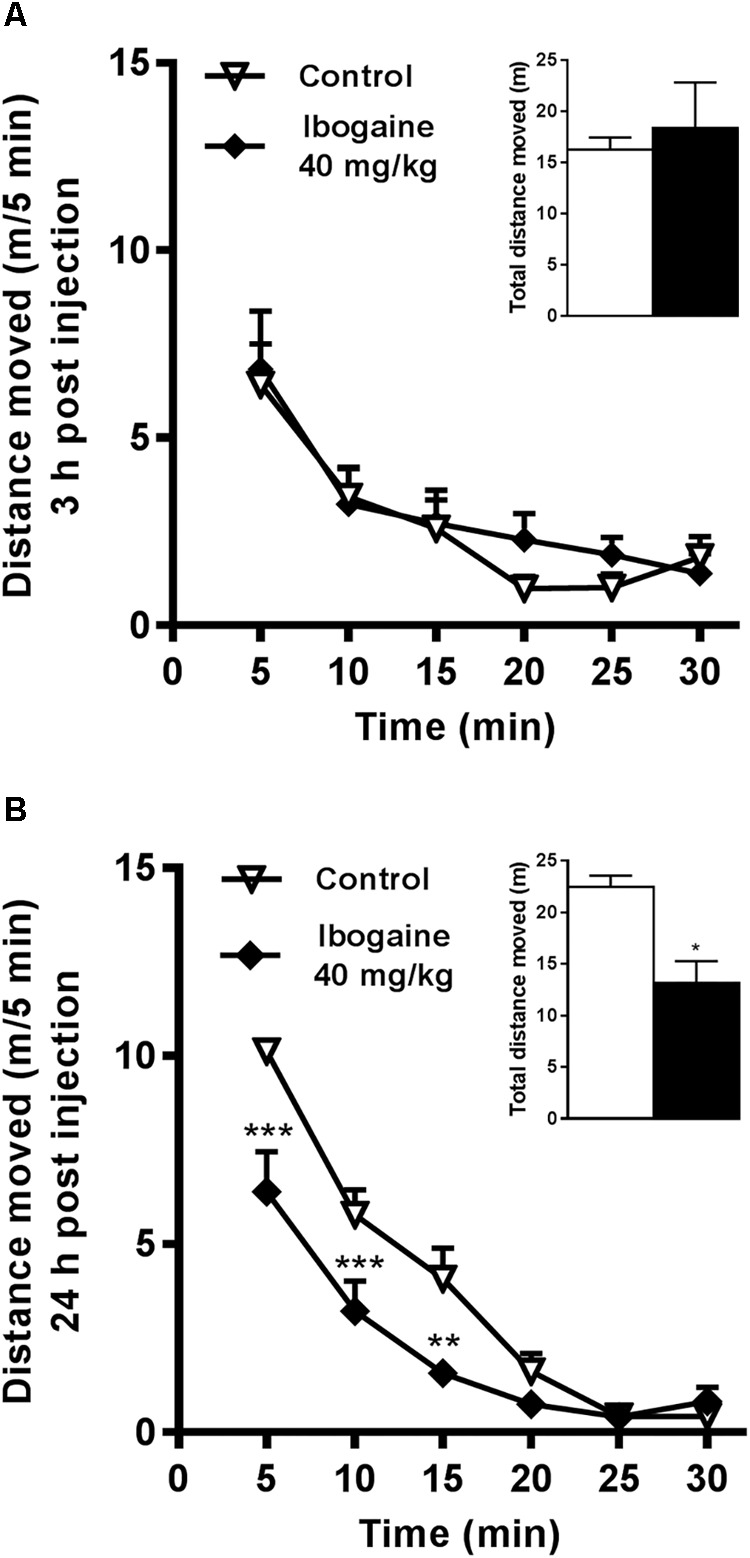
Effects of ibogaine administration on locomotor activity. Locomotor activity of rats was recorded in the OF test during 30 min, at 3 h **(A)** and 24 h **(B)** after ibogaine (40 mg/kg) i.p. administration. The inset graphs represent the total locomotor activity (30 min). Data are expressed as mean + SEM. Data were analyzed by the two-way ANOVA of repeated measured followed by Newman–Keuls test and Unpaired *t*-test (insets). For 24 h after treatment, two-way ANOVA revealed a significant effect of the treatment *F*_(1,8)_ = 11.14, *P* < 0.01, η^2^= 0.059; time *F*_(5,40)_ = 66.56, *P* < 0.001, η^2^= 0.75; and treatment × time interaction *F*_(5,40)_ = 4.85, *P* < 0.01, η^2^= 0.055. ^∗^, respective to saline group. ^∗∗∗^*P* < 0.001; ^∗∗^*P* < 0.01; ^∗^*P* < 0.05. *N* = 18, *n* = 6 per group.

In a previous study, we reported a very high impact of the I_40_ treatment on novelty-induced locomotion after 2 h of ibogaine administration and the concomitant transient induction of some of the behavioral signs related to the serotonergic syndrome (Gonzalez et al., 2018). Thus, we decided to analyze the behavioral effect of ibogaine treatment in the time points used in the present study (3 and 24 h). The behavioral response induced by ibogaine administration is shown in Figure 2. Compared to the control group, novelty-induced locomotion was not altered by I_20_ at any evaluated time (data not shown). Whereas I_40_ was not effective to induce any behavioral alterations 3 h after i.p. administration, it elicited a significantly reduction of the animal locomotion 24 h after injection (Figure 2A,B respectively). No abnormal behaviors were present for both time points and animals were qualitatively indistinguishable from the vehicle group animals (data not shown). Immediately after each behavioral test, animals were sacrificed to pursue brain dissection for the qPCR and Western Blot studies.

### qPCR Quantification of NFs mRNA

**FIGURE 3:**
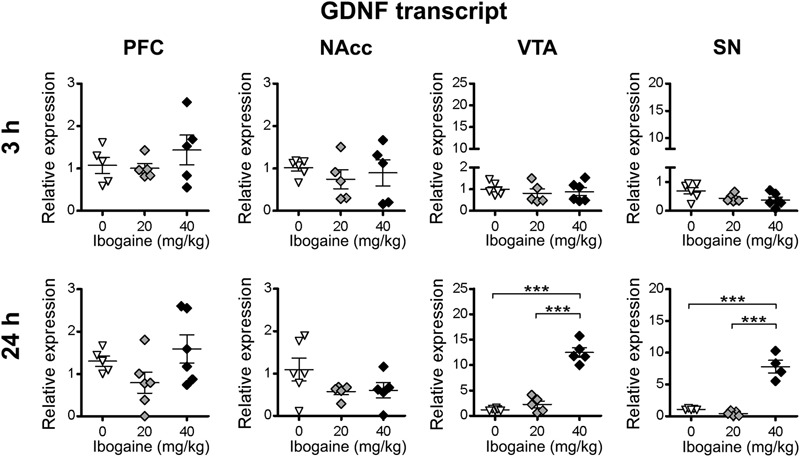
Effects of ibogaine administration on GDNF expression in specific brain areas. Quantitative analysis of GDNF transcript levels in the indicated brain areas after 3 h (upper panels) or 24 h (lower panels) of vehicle (0), 20 or 40 mg/kg ibogaine administration. For 24 h after treatment VTA, *N* = 16, *P* < 0.0001, *F*_2,13_ = 96.11, η^2^ = 0.94; For 24 h after treatment SN, *N* = 14, *P* < 0.0001, *F*_2,11_ = 60.75, η^2^ = 0.92; ^∗∗∗^*P* < 0.001 between indicated groups.

qPCR results for the GDNF (Figure 3) showed that ibogaine acute administration differentially regulated GDNF mRNA expression levels in the selected brain regions in a dose and time-dependent manner. At 3 h, no changes in the GDNF mRNA expression was found for both doses of ibogaine in all the studied areas. In contrast, after 24 h of treatment, changes in the expression of GDNF were found in a dose and site-specific manner. While the I_20_ dose did not affect the GDNF expression in any of the studied areas, the I_40_ dose selectively increased GDNF mRNA content in the midbrain regions: VTA (12-fold increase compared to the control group) and SN (6-fold increase vs. the control group) with no appreciable effects in the PFC and NAcc.

**FIGURE 4:**
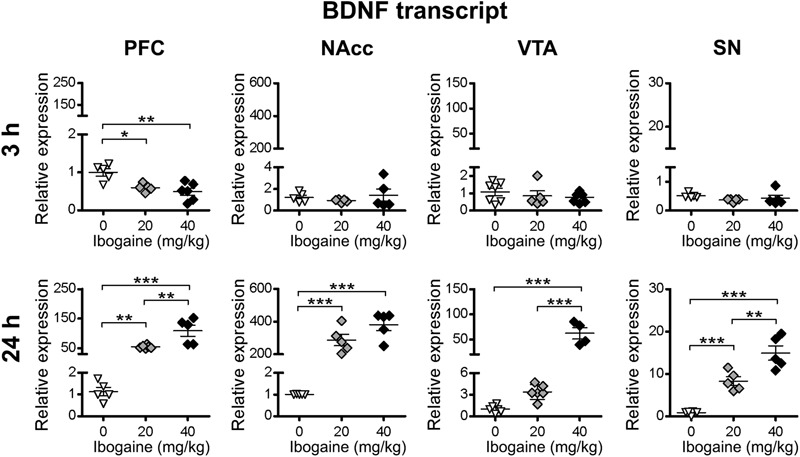
Effects of ibogaine administration on BDNF expression in specific brain areas. Quantitative analysis of BDNF transcript levels in the indicated brain areas after 3 h (upper panels) or 24 h (lower panels) of vehicle (0), 20 or 40 mg/kg ibogaine administration. For 3 h after treatment PFC, *N* = 16, *P* < 0.0001, *F*_2,13_ = 9.80, η^2^ = 0.61; For 24 h after treatment PFC, *N* = 16, *P* < 0.0001, *F*_2,13_ = 25.26, η^2^ = 0.80; For 24 h after treatment NAcc, *N* = 15, *P* < 0.0001, *F*_2,12_ = 46.62, η^2^ = 0.89; For 24 h after treatment VTA, *N* = 14, *P* < 0.0001, *F*_2,11_ = 46.46, η^2^ = 0.88; For 24 h after treatment SN, *N* = 16, *P* < 0.0001, *F*_2,13_ = 45.50, η^2^ = 0.88; ^∗^ *P* < 0.05, ^∗∗^ *P* < 0.01 and ^∗∗∗^*P* < 0.001 between indicated groups.

For BDNF, ibogaine treatment produced an appreciable downregulation of its expression in the PFC at 3 h after injection (1.7 and 2-fold decrease for I_20_ and I_40_, respectively, compared to control, control = 1.000 ± 0.099, I_20_ = 0.596 ± 0.045, I_40_ = 0.492 ± 0.094), while no response was seen for the other brain areas at this time point (Figure 4). At 24 h, ibogaine administration upregulated the mRNA expression of BDNF in all the brain regions studied in a dose-dependent manner (Figure 4). A large effect was found in the NAcc for both doses of ibogaine (220-fold increase compared to the control for I_20_, and 340-fold increase for I_40_). The I_20_ dose increased BDNF expression in PFC (55-fold increase compared to the control) but not in the VTA or SN. On the other hand, in addition to the NAcc, the I_40_ dose also upregulated BDNF expression in PFC (107-fold increase compared to the control), VTA (43-fold increase compared to the control) and SN (21-fold increase compared to the control).

**FIGURE 5:**
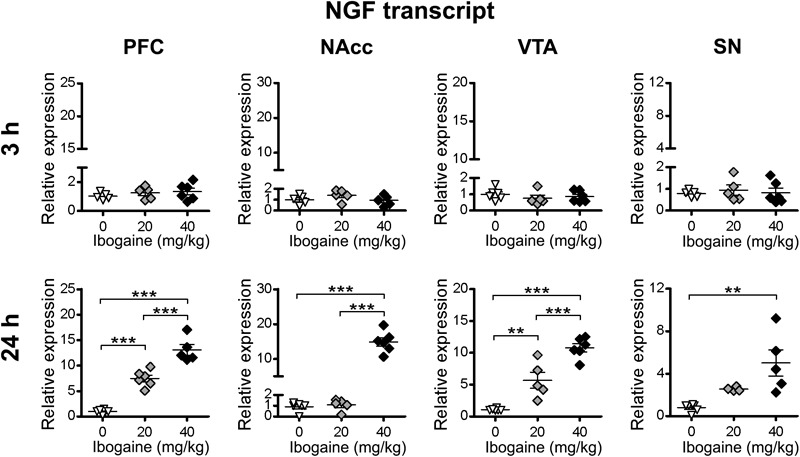
Effects of ibogaine administration on NGF expression in specific brain areas. Quantitative analysis of NGF transcript levels in the indicated brain areas after 3 h (upper panels) or 24 h (lower panels) of vehicle (0), 20 or 40 mg/kg ibogaine administration. For 24 h after treatment PFC, *N* = 17, *P* < 0.0001, *F*_2,14_ = 76.40, η^2^ = 0.92; For 24 h after treatment NAcc, *N* = 17, *P* < 0.0001, *F*_2,14_ = 107.1, η^2^ = 0.94; For 24 h after treatment VTA, *N* = 17, *P* < 0.0001, *F*_2,14_ = 44.88, η^2^ = 0.87; For 24 h after treatment SN, *N* = 16, *P* = 0.0050, *F*_2,13_ = 8.16, η^2^ = 0.61; ^∗∗^*P* < 0.01 and ^∗∗∗^*P* < 0.001 between indicated groups.

For NGF (Figure 5), no difference in the content of mRNA was found 3 h after ibogaine treatments. At 24 h, an upregulation of NGF mRNA content was found in: PFC (14-fold increase compared to the control), NAcc (15-fold increase compared to the control), VTA (11-fold increase compared to the control), and SN (4-fold increase compared to the control). For the I_20_ dose a significant effect was only found in the PFC (7-fold increase compared to the control) and VTA (5-fold increase compared to the control). However, the levels of increase in the NGF mRNA were not as high as those for BDNF.

### GDNF, BDNF and proBDNF Protein Content by Western Blot

**FIGURE 6:**
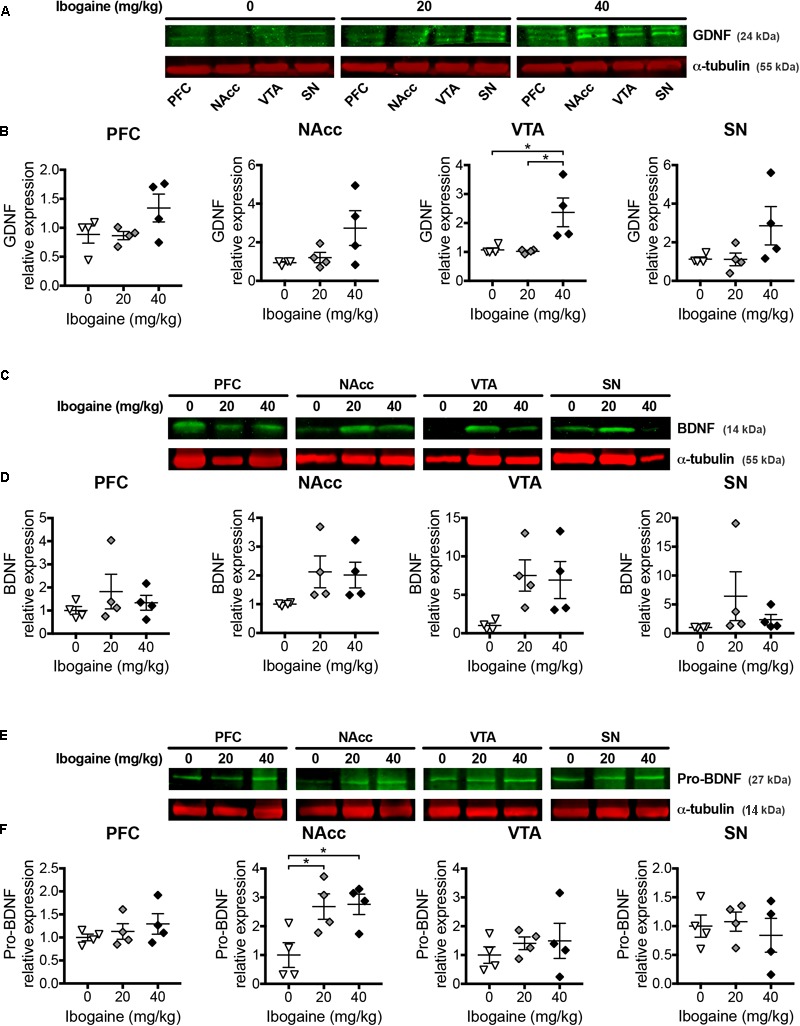
Effects of ibogaine administration on GDNF, BDNF, and proBDNF protein levels in specific brain areas. Western blot analysis of GDNF **(A,B)**, BDNF **(C,D)** and proBDNF **(E,F)** protein levels in the indicated brain areas after 24 h of vehicle (0), 20, or 40 mg/kg ibogaine administration. A representative image from immunostained membrane of each condition is shown **(A,C,E)** with the corresponding quantification below **(B,D,F)**. Data represent mean ± SEM of *n* = 4 biological replicates assayed in triplicate. For GDNF/VTA, *N* = 12, *P* < 0.05, *F*_2,9_ = 6.86, η^2^ = 0.60; For proBDNF/NAcc, *N* = 12, *P* < 0.05, *F*_2,9_ = 5.87, η^2^ = 0.57; ^∗^*P* < 0.05 between indicated groups.

Considering the changes found for the expression of NFs after 24 h of ibogaine administration, we decided to analyze the content of mature proteins BDNF and GDNF for all the studied brain regions, because of their previously mentioned well studied involvement in the addictive behavior. Precursor of BDNF, proBDNF was also considered since it is well described that it shows opposite effects to the mature protein because of a higher affinity to the p75 receptor (Woo et al., 2005; Xu et al., 2011; Sun et al., 2012). For GDNF, a single dose of ibogaine affected mature protein content in a region- and dose-dependent manner (Figure 6). While no changes in GDNF content were observed for I_20_ in any of the studied regions, GDNF content was increased in VTA for the I_40_ dose (2-fold increase compared to the control group). No effect was observed in the GDNF content at the NAcc, SN, and PFC in comparison to the control group. For BDNF no significant change in the mature protein content was detected for all the studied regions for both doses of ibogaine. Nevertheless, in the case of proBDNF we found a selective increase in the protein content for I_20_ and I_40_ in the NAcc (2.7 and 2.8-fold increase for I_20_ and I_40_ doses, respectively, compared to control), while no significant change was detected in the other brain areas.

## Discussion

In the present study, we have demonstrated that ibogaine administration simultaneously alters the transcripts levels of GDNF and BDNF (which have been extensively related to drug-seeking behaviors) in a dose- and time-dependent manner. Additionally, NGF expression was also modified, showing potential effects of ibogaine administration on the expression of other relevant NFs. Regarding the protein content, we showed that after 24 h of treatment, I_40_ selectively increased mature GDNF in the VTA, while proBDNF content was increased selectively in NAcc by both doses. Since as mentioned before, ibogaine is rapidly metabolized to produce noribogaine, further experiments are needed to elucidate if the metabolite and/or the parent drug produced these effects. Considering that dopamine neurotransmission, specifically in the mesocorticolimbic pathway, is related to rewarding/reinforcing and motivational actions of most drugs of abuse (Di Chiara and Imperato, 1988; Koob and Bloom, 1988; Kalivas and Volkow, 2005) our findings contribute to shed light on a mechanism underlying the ability of ibogaine administration to attenuate drug-seeking behavior.

Regarding the motor function, a decrease in the novelty-related motor activity was observed 24 h after I_40_ (while 3 h after the same treatment, animals displayed a similar activity than the control). There is no evidence at this point to establish a potential connection between this intriguing behavior and the observed changes in NFs expression. In this regard, considering the changes in the expression of NFs at 24 h in the SN, it is plausible that a neurochemical imbalance in the basal ganglia output may underlie the changes in the motor activity (Day et al., 2008; Calabresi et al., 2014). Moreover, we cannot rule out that this acute motor impairment is related to this neurochemical effect eliciting a decrease in the animal overall motivation. Behavioral studies using valid and reliable experimental paradigms for studying the effect of ibogaine on reward-related behaviors should be done to understand these observations. On the other hand, we cannot discard the participation of other factors which may be altered in ibogaine-treated animals at this time point.

At 3 h after I_20_ and I_40_ treatments, no alteration of the GDNF transcript content was found in all the studied brain areas. While in a previous report by He et al. (2005), a significant GDNF upregulation was found 3 h after I_40_ treatment in the midbrain of rats, our results show that this increase doesn’t occur in the NAcc and in the specific midbrain areas studied (VTA, NAcc, and SN). On the other hand, after 24 h, we found that the I_40_ dose increased GDNF expression and mature protein content specifically in the rat VTA, which was also found in the whole midbrain at this time point in the mentioned previous report. In this manner our study identifies the VTA as the key brain region of the mesocorticolimbic system where GDNF is upregulated after 24 h of ibogaine administration. This finding is important since the ability of ibogaine to attenuate ethanol self-administration had previously been proposed to be mediated, at least in part, by the increase in GDNF content in the VTA. (He et al., 2005; He and Ron, 2006) Furthermore, we show that I_20_ administration does not increase GDNF expression in any of the studied brain areas, which is in accordance with the observation that this dose was not effective in reducing drug self-administration in the majority of previous studies in rodents (Glick et al., 1991, 1994; Cappendijk and Dzoljic, 1993; Dworkin et al., 1995). In addition, our results are in line with the reports indicating that GDNF infusions into the VTA has been effective in reducing drug self-administration or conditioned place preference for cocaine and alcohol (Messer et al., 2000; He and Ron, 2006; Carnicella et al., 2008, 2009), and with evidence that shows that GDNF mediates negative regulatory effects on chronic morphine-induced neuroadaptations in VTA of rodents (Li et al., 2014; Koskela et al., 2017). Additionally, the selective increase found in this study for GDNF in the VTA by I_40_, could account, at least in part, to the anti-addictive properties of ibogaine considering that upregulation of the GDNF pathway has been proposed a potential strategy to treating SUDs (Carnicella and Ron, 2009). Lastly, I_40_ administration increases GDNF expression in the SN, which was not accompanied with a significant increase of the GDNF protein content at this time point. Given the relevant role of the nigro-striatal pathway in the neuropathology of neurodegenerative disorders like Parkinson ’s Disease (PD) (Dauer and Przedborski, 2003), it would be interesting to study if ibogaine is able to attenuate the cell loss in the SN and the biochemical changes at the striatum throughout the NFs expression using an experimental model of PD.

With regard to BDNF, a selective downregulation of its expression in the PFC for both doses of ibogaine was found after 3 h of administration, while no changes in other areas were observed. Ibogaine and noribogaine administration in rats stimulate the secretion of corticosterone, being ibogaine a more potent releaser (Baumann et al., 2001b). Since corticosterone decreases BDNF expression in the frontal cortex (Dwivedi et al., 2006; Huang et al., 2011), ibogaine induced corticosterone secretion during the first hours after treatment (where ibogaine concentrations in blood are high), could be the reason behind this result. In contrast, at 24 h, an impressive upregulation of BDNF expression was found, which was much more pronounced compared to the effect on GDNF and NGF expression in all the studied brain areas at this time point. Nevertheless, this high effect on BDNF expression was not reflected on an increase in the content of BDNF mature protein (no significant differences were found between both doses and the control group at this time point, although trending toward increased BDNF protein levels in NAcc and VTA for both doses) (Figure 6). Since BDNF is synthesized in a precursor form, we included proBDNF in our experimental design. A selective increase in the proBDNF content was selectively found for NAcc for both ibogaine doses. It is known that the mature BDNF protein and its precursor proBDNF have opposite effects on neuronal protection, axonal growth, maturation of dendrites and synaptic plasticity, owing to different affinities of each form to the TrkB and p75 receptors (Lu et al., 2005; Teng et al., 2005; Benarroch, 2015; Borodinova and Salozhin, 2016; Li et al., 2017). In this regard, since it is well-documented that an increase in BDNF content in the NAcc increases cocaine-seeking behavior (Graham et al., 2007; Bahi et al., 2008) and vulnerability to substance abuse (Krishnan et al., 2007; Burke and Miczek, 2015), an increase in proBDNF in this brain area could have an opposite impact. In this line of reasoning, the increase in proBDNF content in NAcc generated by ibogaine after 24 h of administration in rats could also be implicated in ibogaine’s effect in drug self-administration paradigms. Further experiments are required to address this hypothesis.

Despite implicit assumption that differentially expressed mRNAs are reflected in protein content, numerous previous studies comparing mRNA and protein levels concluded that the correlation is poor (de Sousa Abreu et al., 2009; Maier et al., 2009). While the increase in GDNF mRNA expression was linked to augmented mature protein content, our data showing an impressive increase in BDNF mRNA expression and no changes in mature protein are intriguing. The possibility exists that the time frame of protein synthesis is different for both NFs, however, many other factors should be considered to explain this incongruousness. These include post-transcriptional regulation, for example miRNA-based translation repression or alternative splicing, or translational and post-translational modifications. Indeed, it has been previously described that sortilin, an intracellular chaperon, acts as a regulatory switch for delivery of BDNF to the regulatory secretory pathway or to degradation in the lysosome, modulating in this way the neurotrophic factor availability (Evans et al., 2011). Interestingly, BDNF levels have been shown to be modified in PFC after chronic ethanol exposure (Yang et al., 2017).

How does ibogaine administration produce this long-term upregulation of GDNF and BDNF? It is well established that an increase in serotonin transmission leads to an increase in BDNF expression/signaling both *in vitro* and *in vivo* (Rantamaki et al., 2007; Popova et al., 2017). In addition, serotonin and SSRIs (Selective Serotonin Re-uptake Inhibitors) induce GDNF expression *in vitro* (Hisaoka et al., 2001; Mercier et al., 2004; Tsuchioka et al., 2008; Golan et al., 2011), and recently it has been shown that chronic treatment in mice using SSRIs induce GDNF content in SN and Striatum (Shadfar et al., 2018). It is well-established that ibogaine and noribogaine increase serotonin transmission (Wei et al., 1998; Wells et al., 1999; Baumann et al., 2001b). Both substances are serotonin-reuptake inhibitors (Jacobs et al., 2007; Bulling et al., 2012), and noribogaine is more potent at increasing serotonin levels in the NAcc than ibogaine, which correlates with the ability of both compounds to inhibit SERT *in vitro* (IC_50_ of 3.85 and 0.18 μM for ibogaine and noribogaine, respectively) (Baumann et al., 2001b). In this manner, a sustained enhancement on serotonin transmission due to ibogaine and its long-lasting metabolite noribogaine could account, at least in part, for the observed effect on BDNF and GDNF expression after 24 h of ibogaine administration.

Finally, in addition to GDNF and BDNF, ibogaine also modulated the expression of other NF as NGF, 24 h after treatment, while no changes were found at 3 h. The effect of NGF administration in specific brain areas on drug-seeking behavior has been much less studied in comparison to GDNF and BDNF, and scarce data is available on the effects of NGF in brain regions related to the dopaminergic mesocorticolimbic circuitry. Nevertheless, NGF (as other neurotrophins) is likely involved in mediating important responses related to chronic intake of drugs of abuse, as illustrated by previous studies that show that NGF content decreases in the hippocampus and hypothalamus of alcohol-treated mice (Aloe et al., 1993) and in the serum of chronic heroin and cocaine users (Angelucci et al., 2007). Also, NGF administration into the central nucleus of the amygdala mimicked the morphine reward sensitization (Bie et al., 2012).

The modifications found in NFs levels induced by ibogaine/noribogaine, may underlie neuroplasticity processes in the discrete brain regions analyzed as has been described by several drugs used in clinical practice including drugs of abuse (Castren and Antila, 2017). Most of these drugs regulate the expression of NFs, reactivating a process defined as induced plasticity (iPlasticity), which allows networks reorganization in the adult brain (Castren and Antila, 2017). This is in accordance with the fact that recently noribogaine has been recently classified as a “psychoplastogen,” since it is capable to promote neuritogenesis in cultured rat cortical neurons (Ly et al., 2018). In this manner, neuroplastic changes generated by the selective increase in NFs expression after ibogaine administration could explain, at least in part, the ability of ibogaine to attenuate drug-seeking behavior in rodents (which could be related to its effects on drug craving and reinstatement in humans).

## Conclusion and Future Perspectives

This study demonstrates for the first time that ibogaine administration simultaneously alters the expression of GDNF, BDNF, and NGF transcripts in rat brain regions related to the dopamine neurotransmission in a dose- and time-dependent manner. Our results add relevant information concerning specific brain areas involved in the increment of GDNF levels (VTA) as a putative mechanism of action underlying the anti-addictive effect of ibogaine. In addition, we showed that only I_40_ promoted this increase in GDNF content, which is in accordance with previous reports where the I_20_ treatment was not effective in reducing drug self-administration in rodents (Glick et al., 1991, 1994; Cappendijk and Dzoljic, 1993; Dworkin et al., 1995). Also, we found that both doses of ibogaine produced an increase in the proBDNF content in NAcc after 24 h of treatment, which could be another factor mediating long-lasting effects of ibogaine related to attenuate drug dependence, in addition to the already highlighted increase in GDNF. Future experiments are needed to clarify these important implications in order to elucidate ibogaine’s biological mechanism to attenuate drug seeking behavior. Considering safety concerns raised by adverse effects found in humans after ibogaine intake, such as prolongation of the QT_C_ interval in the EKG (which has been associated with sudden death cases after ibogaine intake) (Koenig and Hilber, 2015), contributions to the understanding of ibogaine’s mechanism of action will provide basis for the preparation of safer and more effective analogs in the future.

## Author Contributions

SM, BG, and SR-B performed the qPCR experiments. EM and SR-B performed the Western Blot experiments. SM, BG, LM-P, and MP contributed in the experiments and analyzed the data. BG prepared the ibogaine HCl used in this study. JP, PR, and CS performed the experiments with animals and the brain dissection. IC, PC, GS, and CS provided the funding for the experiments. MP, PC, GS, CS, DS, and IC planned the experiments and wrote the manuscript. All the authors participated in critical revision the manuscript, added important intellectual content, and approved the definitive version.

## Conflict of Interest Statement

The authors declare that the research was conducted in the absence of any commercial or financial relationships that could be construed as a potential conflict of interest.
