# Supplementary material for: Ibogaine Administration Modifies GDNF and BDNF Expression in Brain Regions Involved in Mesocorticolimbic and Nigral Dopaminergic Circuits
Source: Front Pharmacol. 2019 Mar 5;10:193. doi: 10.3389/fphar.2019.00193 (PMC6411846; doi:10.3389/fphar.2019.00193)
Supplement: Supplementary file 1 [file Data_Sheet_1.pdf]

Supplementary Material

Ibogaine administration modifies GDNF and BDNF expression in brain regions involved in mesocorticolimbic and nigral dopaminergic circuits.

Soledad Marton†, Bruno González†, Sebastián Rodríguez, Ernesto Miquel, Laura Martínez Palma, Mariana Pazos, José Pedro Prieto, Paola Rodríguez, Dalibor Sames, Gustavo Seoane, Cecilia Scorza*, Patricia Cassina*, Ignacio Carrera*

***Correspondence: Cecilia Scorza (cscorza@iibce.edu.uy), Patricia Cassina (pcassina@fmed.edu.uy), Ignacio Carrera (icarrera@fq.edu.uy)**

Supplementary Data

1.1 Ibogaine-HCl Preparation. Nuclear Magnetic Resonance spectra were obtained on a Bruker Avance DPX-400 instrument. In both cases samples were characterized as voacangine or ibogaine according to the following NMR data

Voacangine (12-methoxy-16-carbomethoxyibogamine) isolated from *Voacanga Africana* root bark (see Material and Methods section in the manuscript)
^{1}H NMR (400 MHz, $CDCl_3$) δ 7.81 (s, 1H), 7.13 (d, *J* = 8.7 Hz, 1H), 6.93 (d, *J* = 2.3 Hz, 1H), 6.80 (dd, J = 8.7, 2.4 Hz, 1H), 3.84 (s, 3H), 3.70 (s, 3H), 3.55 (s, 1H), 3.44 – 3.33 (m, 1H), 3.26 – 3.08 (m, 2H), 3.03 – 2.86 (m, 2H), 2.81 (d, *J* = 8.5 Hz, 1H), 2.58 (dd, *J* = 11.8, 2.1 Hz, 1H), 1.94 – 1.81 (m, 2H), 1.73 (t, *J* = 11.1 Hz, 1H), 1.56 (dt, *J* = 22.0, 7.4 Hz, 1H), 1.49 – 1.39 (m, 1H), 1.37 – 1.28 (m, 2H), 1.15 – 1.08 (m, 1H), 0.90 (t, *J* = 7.3 Hz, 3H). **^{13}C NMR** (100 MHz, $CDCl_3$) δ 175.71, 153.98, 137.51, 130.53, 129.19, 116.62, 111.81, 111.07, 110.12, 100.77, 99.99, 77.34, 77.03, 76.71, 57.52, 56.04, 55.14, 53.13, 52.59, 51.52, 39.15, 36.56, 32.03, 27.35, 26.76, 22.22, 11.68.

Ibogaine·HCl prepared by decarboxylation of voacangine (see Material and Methods section in the manuscript)
^{1}H NMR (400 MHz, CD_3OD) δ (ppm) =7.19 (d, *J* = 8.9 Hz, 1H), 6.97 (d, *J* = 2.4 Hz, 1H), 6.76 (dd, *J* = 8.8, 2.5 Hz, 1H), 3.87 (s, 3H), 3.70 (dt, *J*= 13.4, 4.2 Hz, 1H), 3.63 – 3.53 (m, 2H), 3.45 – 3.34 (m, 3H), 3.31 – 3.14 (m, 2H), 2.32 (ddt, 13.5, 12.1, 2.7 Hz, 1H), 2.19 – 2.09 (m, 2H), 2.06 (hept, *J* = 7.5 Hz, 1H), 1.74 – 1.65 (m, 3H), 1.46 – 1.34 (m, 1H), 1.03 (t, *J* = 7.3 Hz, 3H) **^{13}C NMR** (100 MHz, CD_3OD) δ(ppm) = 153.0, 139.1, 130.4, 128.5, 111.2, 111.1, 106.0, 99.5, 60.1, 56.0, 54.9, 50.5, 39.0, 35.1, 31.2, 28.8, 26.0, 23.9, 18,0, 10.5

To determine ibogaine-HCl purity (after crystallization and purification procedures described in the Materials and Methods section of the manuscript) Gas Chromatography analysis was carried out in a GC-MS Shimadzu QP 1100 EX instrument using the electron impact mode, 70 eV. For analysis sample was previously dissolved in aqueous NaOH 10% and extracted with Ethyl Acetate. Conditions: Column HP-5MS (30m x 0.25mm x 0.25um) Temperature Program 200 ºC (Hold time, 2 minutes) to 300 ºC (Hold time, 5 minutes) with a rate of 10 ºC/min. Ibogaine purity was determined as 98.3% (See chromatogram below)

2 Supplementary Figures and Tables

2.1 Supplementary Figures

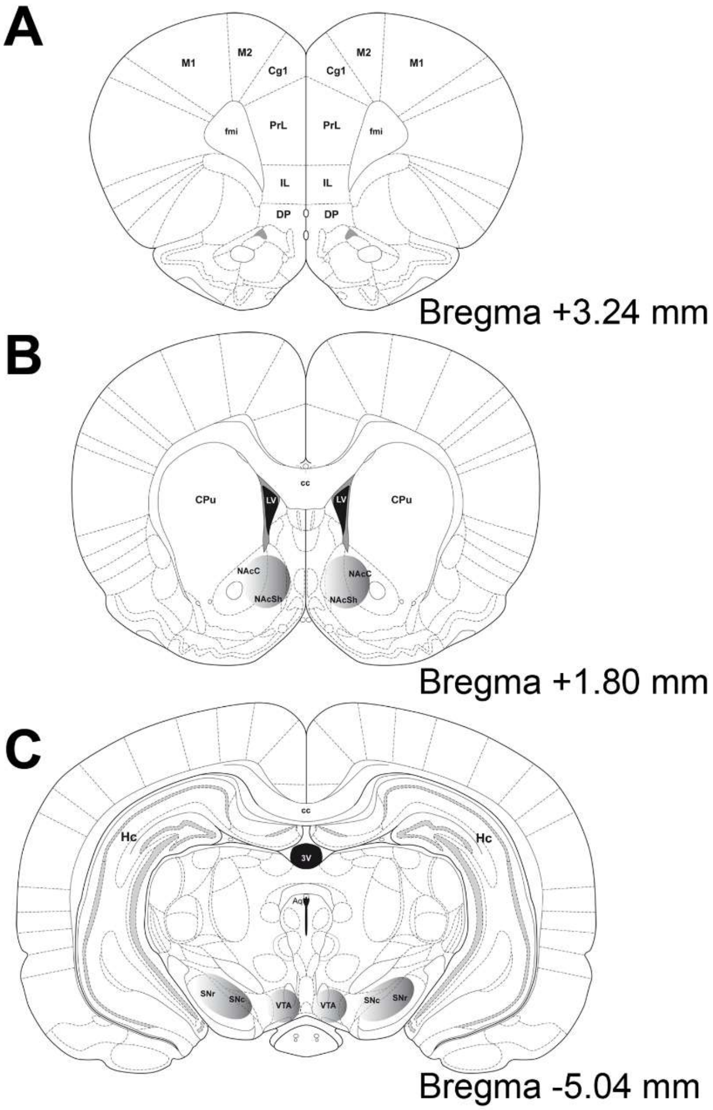

Supplementary Figure 1. Dissected brain areas. Figure shows a schematic diagram of coronal sections at three different levels. A: +3.24 for PFC, B: + 1.80 for NAcc, and C: -5.04 for VTA and SN, from Bregma) according to Paxinos and Watson (2005) atlas. In A, a complete slice (2 mm) was taken for PFC (including the mPFC). In B and C, gray areas indicate an approximately extension of the brain tissue dissected for NAcc ((this region was punched by using a sample corer with an inner diameter of 2 mm), VTA and SN, respectively. Cg, cingulate cortex; PrL, prelimbic; IL, infralimbic; M1 and M2, motor cortex; CPu, corpus striatum; NAcC and NAcSh, nucleus accumbens core and shell; Hc, hippocampus; CC, corpus callosum; 3V, third ventricle, SNc and SNr, compact and reticular subtantia nigra; VTA, ventral tegmental area.

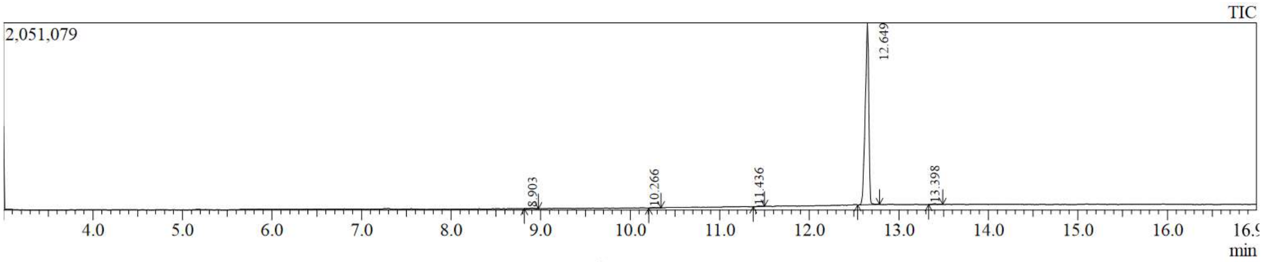

Peak Report TIC

Peak#	R.Time	I.Time	F.Time	Area	Area%	Height	Height%	A/H	Mark	Name
1	8.903	8.817	8.975	32164	0.58	9633	0.48	3.34	MI	
2	10.266	10.208	10.342	16222	0.29	6610	0.33	2.45	MI	
3	11.436	11.375	11.500	16385	0.30	6125	0.30	2.68	MI	
4	12.649	12.542	12.783	5421252	98.31	1990692	98.49	2.72	MI	
5	13.398	13.333	13.492	28586	0.52	8238	0.41	3.47	MI	
				5514609	100.00	2021298	100.00			

Supplementary Figure 2. Ibogaine-HCl GC-MS chromatogram.

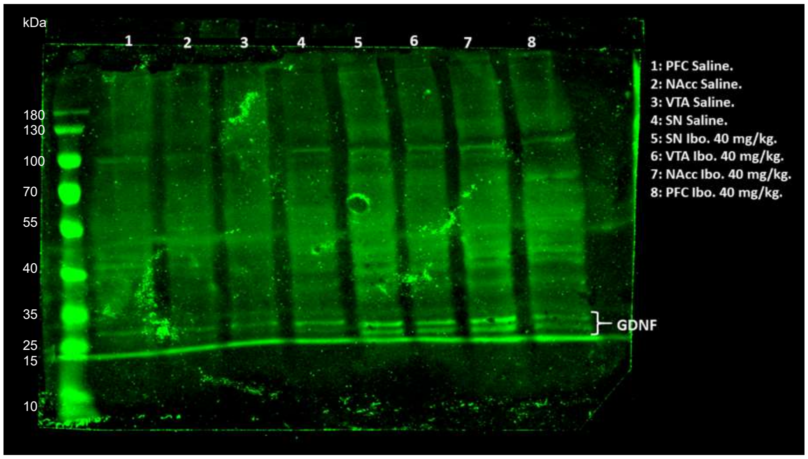

Supplementary Figure 3. Nitrocelulose membrane incubated with GDNF antibody, for Saline and Ibogaine 40 mg/kg samples.

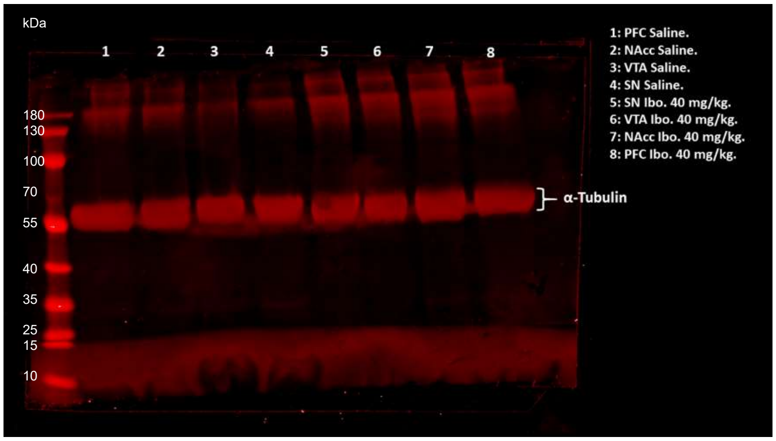

Supplementary Figure 4. Nitrocelulose membrane incubated with alpha-tubulin antibody, for Saline and Ibogaine 40 mg/kg samples.

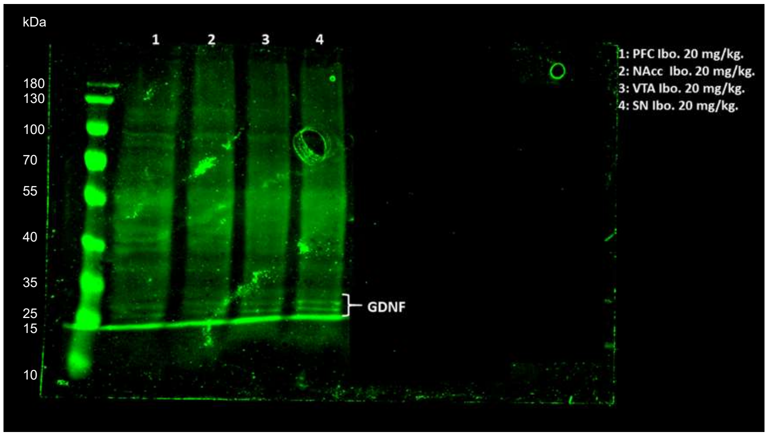

Supplementary Figure 5. Nitrocelulose membrane incubated with GDNF antibody, for Ibogaine 20 mg/kg samples.

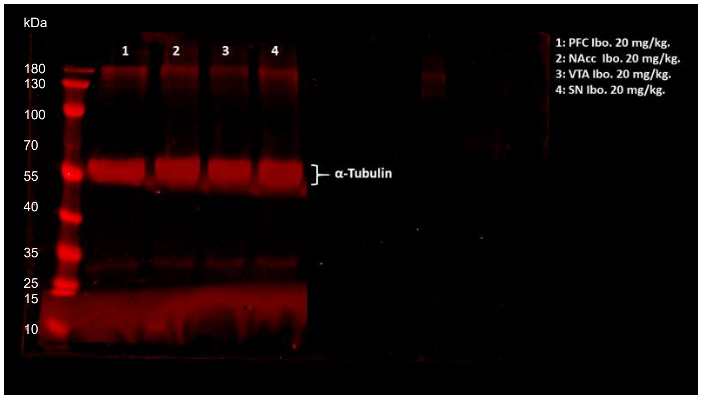

Supplementary Figure 6. Nitrocelulose membrane incubated with alpha-tubulin antibody, for Ibogaine 20 mg/kg samples.

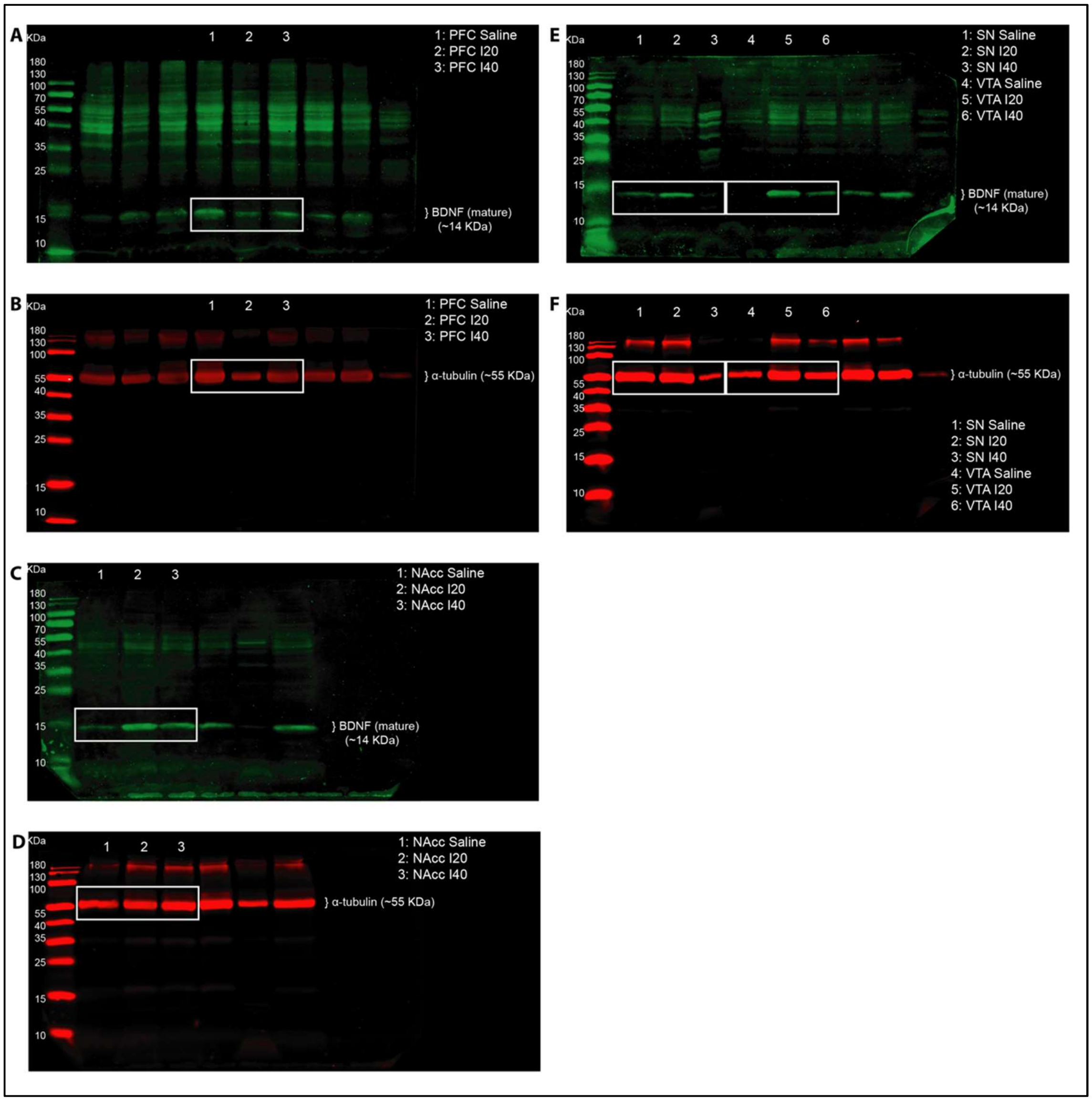

Supplementary Figure 7. Full-scanned images of western blots in main Figure 7-C. Nitrocellulose membranes corresponding to samples of PFC (**A-B**), NAcc **(C-D)**, VTA and SN **(E-F)** from saline, I20 and I40-treated rats incubated with antibodies to BDNF **(A, C, E)** and α-tubulin **(B, D, F)**.

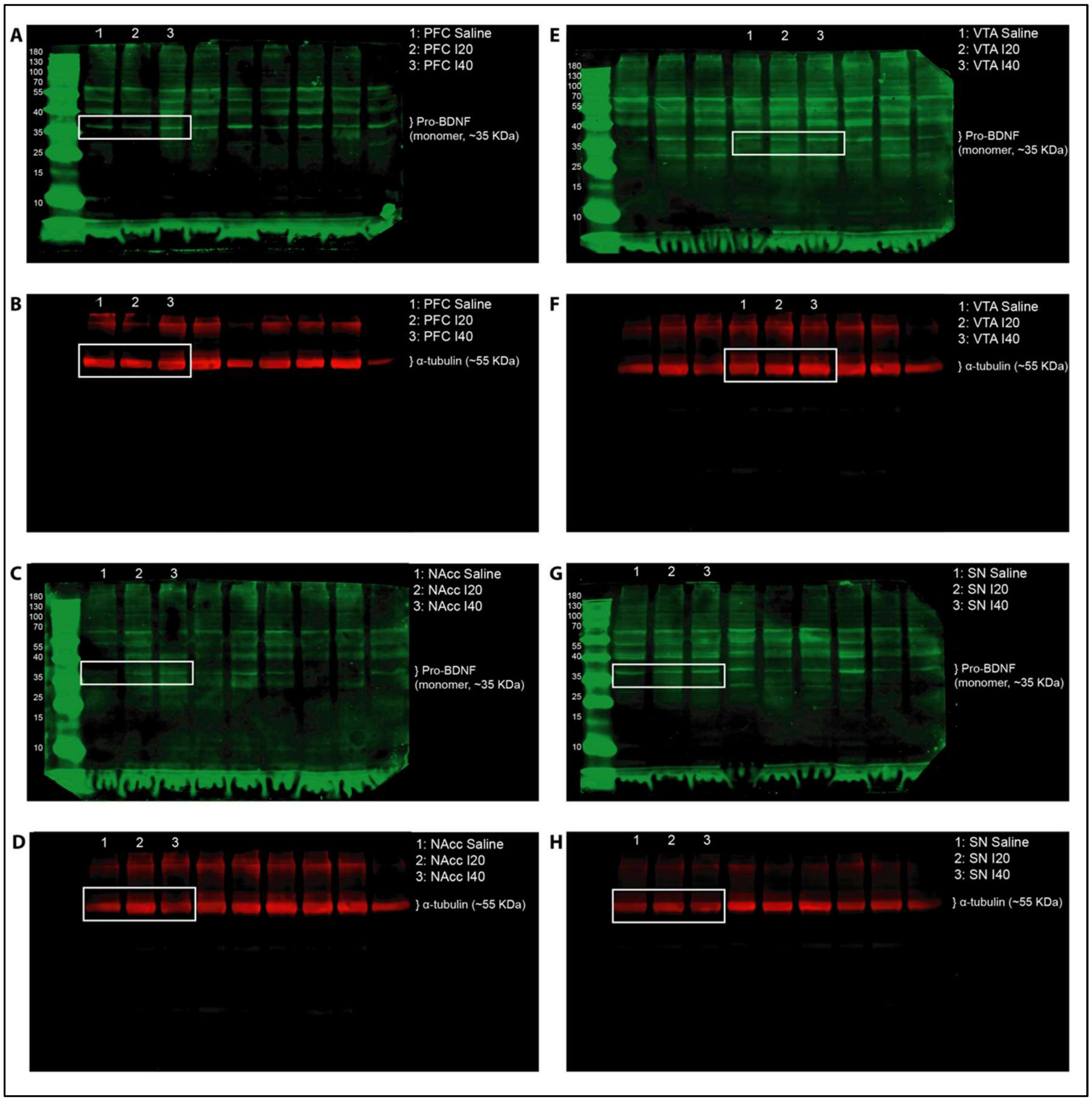

Supplementary Figure 8. Full-scanned images of western blots in main Figure 7-E. Nitrocellulose membranes corresponding to samples of PFC (**A-B**), NAcc **(C-D)**, VTA **(E-F)** and SN **(G-H)** from saline, I20 and I40-treated rats incubated with antibodies to ProBDNF **(A, C, E, G)** and α-tubulin **(B, D, F, H)**.
